# Technical note: A comparison between rehydrating solutions in the pretreatment of mummified and corified skin for forensic microscopic examination

**DOI:** 10.1007/s00414-022-02833-x

**Published:** 2022-05-11

**Authors:** Stefano Tambuzzi, Guendalina Gentile, Gianluigi Bilardo, Michele Boracchi, Paolo Bailo, Teresa Casalino, Salvatore Andreola, Riccardo Zoja

**Affiliations:** 1 Laboratorio di Istopatologia Forense e Microbiologia Medico Legale, Sezione di Medicina Legale e delle Assicurazioni, Dipartimento di Scienze Biomediche per la Salute, Università degli Studi di Milano Via Luigi Mangiagalli, 37, 20133 Milan, Italy; 2 Università degli Studi di Camerino, Camerino, Italy

**Keywords:** Forensic pathology, Mummification, Corification, Sandison’s rehydrating solution, Fabric softener, Body lotion

## Abstract

Microscopic examination of mummified or corified skin may be of extreme importance for forensic purposes. However, standard histological samples in these cases are low-end, and preparation is burdened by several problems and so are diagnostic results: an improvement of these types of specimens is therefore advantageous. This study aims to identify the best performing rehydration solution among a fabric softener, a body lotion, and Sandison’s rehydrating solution. Samples of skin undergoing mummification or corification were collected from 25 corpses and each sample was divided into 4 fragments: one of these fragments was directly fixated in 4% formalin, one was previously treated with a tissue softener, another one was previously treated with a body lotion, and the last one was treated with Sandison’s solution. After 72 h, the pretreated samples were post-fixated in 4% formalin and then prepared for standard histological examination staining the histological slides with hematoxylin-eosin and Masson’s trichrome. At the microscopic examination, samples directly fixated in formalin were characterized by usual marked structural alterations and altered stainability, typical of such dry tissues. Vice versa, those previously treated appeared to be better-preserved even though with different improvement levels: body lotion made a medium–low-grade restoration of the tissues, and fabric softener a high-grade restoration, while Sandison’s rehydrating solution produced an optimal grade restoration. Sandison’s rehydrating solution was confirmed to be the best rehydrating substance for mummified and corified skin. Fabric softener could be, however, considered a valid substitute, being productive of high-grade microscopic yield.

## Introduction

Under peculiar climatic and environmental conditions, cadaveric putrefaction stops [1] and “conservative” transformative processes [2] such as mummification and corification [3] take place. Responsible for these two phenomena are both the percentage of humidity of the place in which the body remains after death [4] and the intrinsic conditions of the corpse [5]. Complete mummification and corification usually require several months to realize, although such phenomena can affect, at much earlier times, focal areas of skin, especially the acromial extremities, with high interindividual variability [2]. It is very common that, after the exhumation or the discovery of a mummified or corified corpse, a judicial autopsy might be required, possibly followed by histopathological investigations [6]. However, in such cases, the histological examination assessed with standard preparation can be very difficult or impossible to be performed. Many negative effects can be experienced in all the phases of the histological preparation, due to the intrinsic hardness and dryness of the tissues, their altered stainability, and possible loss of material [7]. In these cases, it could be very difficult to distinguish the “real pathology” and the possible crucial forensic aspects of wounds from the “post-mortal artifacts” that often overlap each other [8]. Consequently, it becomes essential to have effective improving histological procedures, such as rehydration techniques. To date, forensic literature identifies Sandison’s rehydrating solution as the best choice for restoring highly degraded skin, such as mummified or corified tissues [9]. Isolated application of comfort fabric softener on Egyptian mummies has also been described in the 1980s [10].

The authors tested the efficacy of two innovative rehydrating solutions on mummified and corified corpses: a fabric softener and a body lotion, both commercially used. The effectiveness of these two substances has been evaluated on the basis of their microscopic performance, comparing them to each other, with the Sandison’s rehydration solution and with the direct fixation in formalin. The aim was to identify the most performing one or a valuable alternative, in order to obtain better histological preparations, facilitate forensic diagnoses, and provide forensic pathologists a guideline to follow when facing microscopic examination of these peculiar and insidious cadaveric tissues.

## Materials and methods

### Selection of the case series

We considered 25 corpses undergoing autopsy, in a state of mummification or corification process. Fourteen were male and 11 were female, with an age range from 19 to 90 years. Seventeen of the subjects died of natural causes, 1 of suicide, 1 of traffic accident, 1 of accidental event, and 5 of homicide. In most cases, the victims were found at home, with a few exceptions. Two cases (no. 1 and no. 18) were exhumed corpses, and one case (no. 7) was a traffic accident victim who had undergone an initial conservation process while in cold storage. Post-mortem intervals (PMI) refer to the time elapsed between the autopsy and the last time the victims were seen or heard alive. Cases were selected on the basis of macroscopic observation of mummified or corified skin areas: we considered mummified skin the one that appeared yellow-brownish colored, dry with a hard consistency, parchment-like, and similar to old-tanned leather; conversely, we considered corified the skin that appeared grayish colored, quite hard and elastic, becoming similar to recently tanned leather. Using a scalpel, a skin fragment sized 2.0 cm × 2.0 cm × 0.5 cm was sampled from each corpse during the autopsy. We need to point out that case no. 10 was a murder accomplished with a stabbing weapon, in which the corified area was sampled in close proximity to a wound margin. Also, in case no. 21, the skin sample was characterized not only by mummification but also by partial carbonization, since the victim’s body was burnt and found 5 months later. Table 1 shows the details of the enrolled cases.

**Table 1:** Presentation of general data from the 25 cases enrolled for the study

Case no.	PMI	Gender	Age	Preservation state of the analyzed skin fragment	Manner of death	Skin sampling site
1	9 months	F	87	Mummified	Homicide (poisoning)	Left periumbilical
2	4 months	M	89	Corified	Accident (CO intoxication)	Right anterior neck
3	10 days	M	89	Corified	Natural death	Periumbilical
4	14 days	M	90	Corified	Natural death	Right knee
5	5 days	F	48	Corified	Suicide (drug intoxication)	Left parasternal
6	3 days	F	49	Corified	Natural death	Right thigh
7	14 h	M	27	Corified	Traffic accident (motorbike against car)	Left knee
8	3 days	F	49	Corified	Natural death	Left anterior chest
9	3 days	F	77	Corified	Natural death	Left dorsal
10	6 days	F	19	Corified	Natural death	Right thigh
11	4 days	M	28	Corified	Natural death	Right knee
12	9 months	M	70	Mummified	Natural death	Right chest
13	2 months	F	80	Mummified	Natural death	Right knee
14	10 days	M	73	Corified	Natural death	Left hand
15	6 months	M	42	Mummified	Natural death	Right foot
16	2 months	M	83	Mummified	Natural death	Left foot
17	1 month	F	62	Mummified	Natural death	Right leg
18	3 years	M	78	Mummified	Homicide (poisoning)	Right back
19	21 days	F	39	Corified	Homicide (blunt force)	Left chest
20	3 months	M	81	Mummified	Natural death	Right abdomen
21	5 months	F	38	Mummified	Homicide (strangulation and charring)	Right abdomen
22	40 days	M	86	Corified	Natural death	3rd finger right hand
23	2 months	M	47	Mummified	Natural death	3rd finger left hand
24	4 months	F	39	Mummified	Natural death	Right hand
25	3 days	M	53	Corified	Natural death	Left back

### Procedure for preparing cadaveric samples for rehydration

After collection, each fragment of mummified or corified skin was divided into four equal portions (1-2-3-4), each treated differently:

- Portion 1 of the sample was directly fixated in 4% formalin produced by *Bio Optica®* (CAS No. 50-00-0);
- Portion 2 of the sample was pretreated with the commercial fabric softener “*Coccolino ammorbidente liquido*”*®* produced by *Unilever* (CAS N.68500400) composed of *benzisothiazolinone* (antibacterial and antifungal), *alpha-iso methyl ionone*, *butylphenyl methylpropional*, *amyl cinnamal* (aromas), *ethanone1*, *triethanolamine dialkyl ester* (cationic surfactants), *triethanolamine methylsulfate* (pH balancer), *unsaturated fatty acids C10-20*, and *C16-18* (emollients);
- Portion 3 of the sample was pretreated with the commercial body lotion “*Nivea Nourishing Body Lotion*”*®* produced by *Beiersdorf AG group* (CODE 80212) consisting of *aqua*, *paraffinum liquidum*, *isohexadecane*, *glycerin*, *isopropyl palmitate*, *PEG-40 Sorbitan Perisostearate*, *microcrystalline wax*, *polyglyceryl-3 diisostearate*, *Prunus amygdalus dulcis oil*, *tocopherol*, *magnesium sulfate*, *sodium citrate*, *citric acid*, *tocopheryl acetate*, *potassium sorbate*, *linalool*, *limonene*, *benzyl alcohol*, *geraniol*, *citronellol*, *alpha-isomethyl ionone*, *benzyl benzoate*, *parfum*, *almond oil*, *vitamin E*;
- Portion 4 of the sample was pretreated with Sandison’s rehydration solution consisting of *20 ml of 5% sodium carbonate Na*_*2*_*CO*_*3*_, *30 ml of 96% ethanol*, and *50 ml of 1% formalin*.

**Fig. 1:**
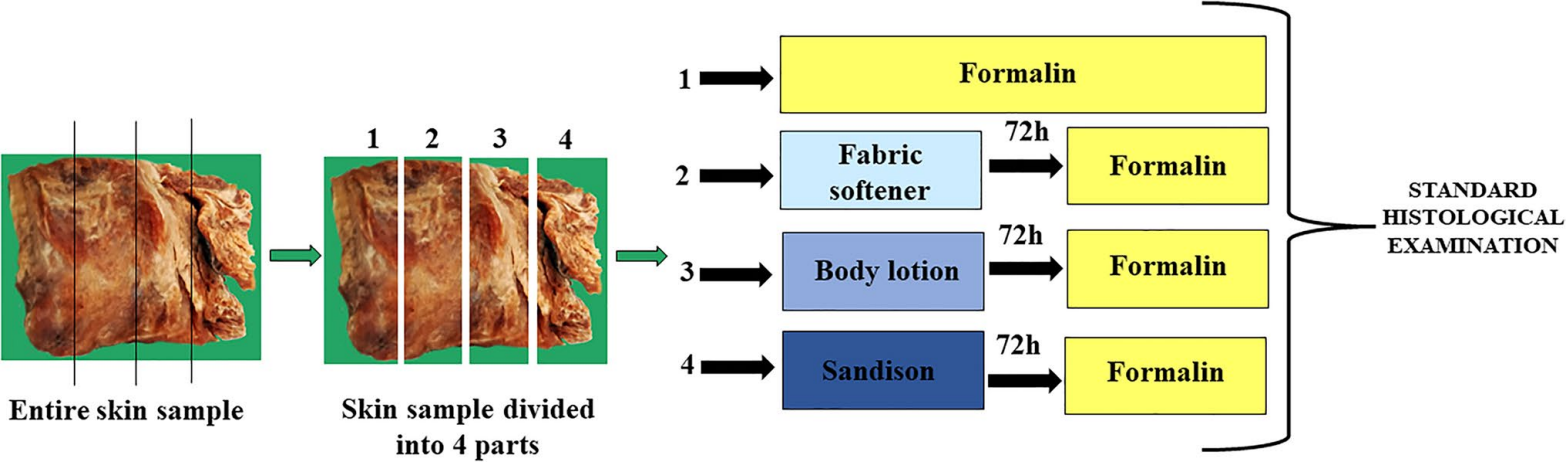
Laboratory procedure scheme performed on each skin sample

Skin samples were placed in body lotion and fabric softener for 72 h, a period of time currently used for the standard pretreatment with Sandison’s solution. Subsequently, all samples were fixated in 4% formalin according to the scheme shown in Fig. 1.

### Procedure for preparation of cadaveric specimens for standard histological examination

At the end of the 4% formalin fixation phase, all the 100 skin fragments (25 fixated directly in formalin, 25 pretreated with fabric softener, 25 pretreated with body lotion, and 25 pretreated with Sandison’s solution) were prepared for standard histological examination. The samples were dehydrated in increasing ethanol solutions, clarified in xylene substitute - Microscopy Neo-Clear® WVR (CAS No. 1330-20-7), and embedded in high melting point (56–58C°) paraffin - Paraplast WVR (CAS No. 64742-51-4). From each paraffin-embedded fragment, we cut out 2 twin sections of 5-μm-thick slides by means of a Reichert-Jung Pabisch model OME slide microtome and Microtome blade stainless steel N35H Feather ©; each slide was loaded on a Super Frost® glass slide holder and dried in a thermostatic chamber at a constant temperature of 37°C for 5 days. We then stained one section with hematoxylin-eosin (H&E) and the other one with Masson-Goldner’s trichrome (TM) technique. The 200 slides obtained were observed using a Leica DMR microscope and the most significant images were photographed with a Leica DC 300F digital camera.

## Results

The application of different rehydrating solutions to mummified and corified cadaveric skin provided interesting macroscopic and microscopic results.

### Macroscopic characteristics of skin fragments

Before the histological preparation procedure, mummified skin fragments had markedly hard consistency: mummified samples were parchment-like and brownish-yellow colored, with the sole exception of the partially charred one, which was black-brownish colored; the corified fragments, however, were characterized by a hard-elastic consistency and a grayish color.

The skin samples directly fixated in formalin, once extracted from the solution, appeared rigid, and retracted, with a very hard consistency but, at the same time, fragile, with an easy detachment of dry portions.

After pretreatment with body lotion, fabric softener, and Sandison’s rehydration solution, skin samples appeared smooth, stretched, silky, and with a soft consistency. These characteristics were particularly noticeable in the specimens pretreated with fabric softener and Sandison’s solution, which also made the specimens lighter in color by removing their brownish pigmentations. After the formalin fixation phase, we observed that only the skin samples pretreated with the body lotion had lost most of their previously acquired softness returning to hard consistency.

### Microscopic characteristics of sections obtained by direct fixation in formalin 4%

**Fig. 2:**
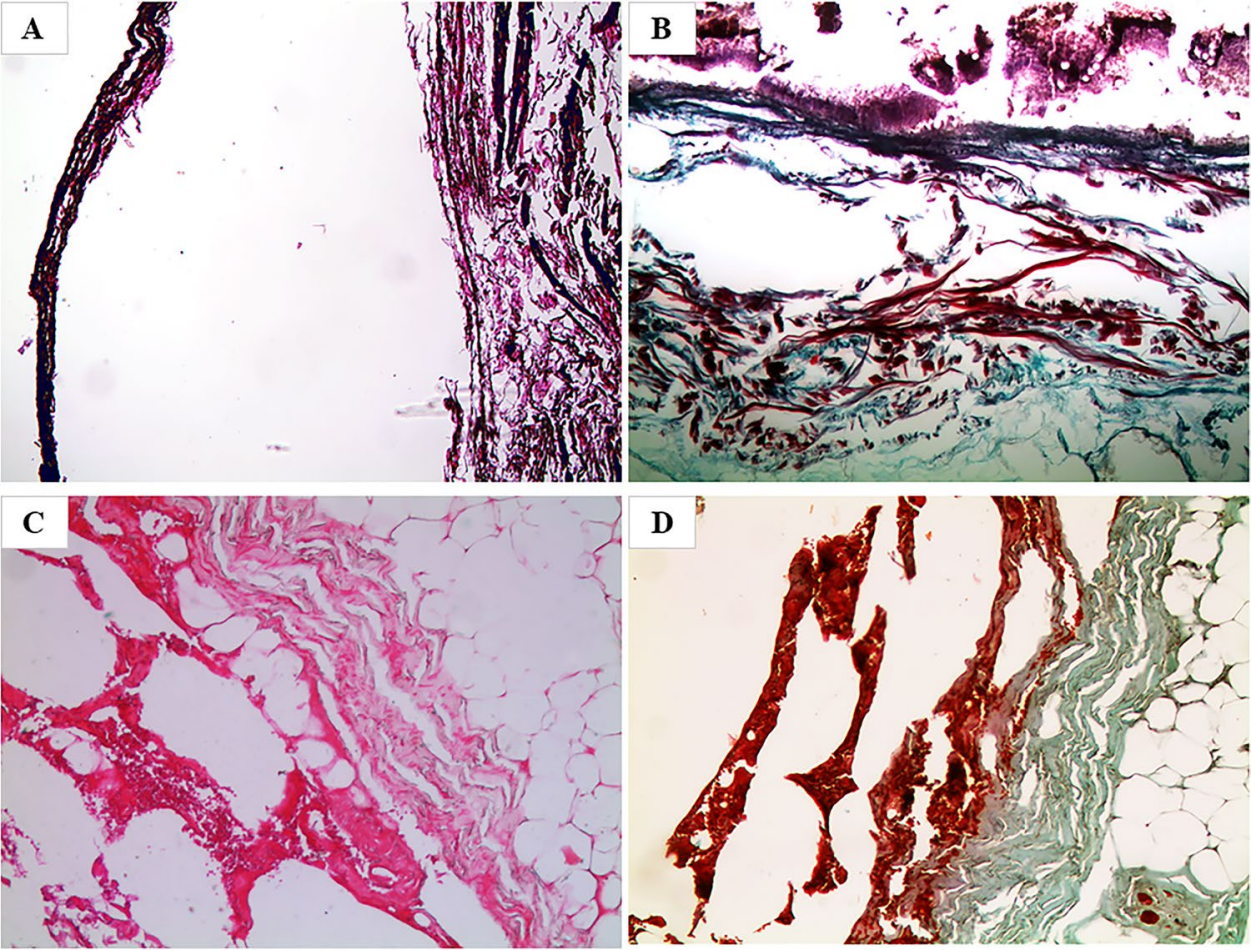
Microscopic view of preparations obtained after direct fixation in 4% formalin with marked structural alterations and altered stainability of the different tissue portions caused by severe and persistent drying. **A** Case no. 4 (H&E, 50×). **B** Case no. 6 (TM, 50×). **C**, **D** Case no. 10 (H&E, 100×, and TM, 100×, respectively) with evidence of vital hemorrhagic infiltrates

All the samples stained with H&E showed structural alterations of the different tissue portions with marked alterations of the dyeing affinity (Fig. 2A) and marked deterioration effects with confirmation of the severe degree of desiccation. The cutaneous structure was no longer homogeneous and there were gross losses of substance, with wide areas of breakage and subversion of the structures that, in some areas, overlapped each other. The epithelium was diffusely de-epithelialized with the presence of fungal and bacterial colonies from putrefaction on the surface of the samples; the dermis showed marked degenerative post-mortal aspects and, in the subcutaneous adipose tissue, there was the occasional finding of residues of elastic and collagen arterial parietal fibers; in the vessels, the presence of eosinophilic granular material, ascribable to blood cells in a high degree of autolysis, could be observed. Twin sections, stained with TM, showed similar structural and stainability alterations, with the dermis and adipose tissue being reddish and granular in appearance, rather than green as usual (Fig. 2B). In case no. 10, referred to the homicide accomplished with a stabbing weapon and the subsequent corification of the victim, the sample analyzed and directly fixated in formalin allowed to observe both with H&E (Fig. 2C) and TM (Fig. 2D) the presence of vital hemorrhagic infiltrates, localized in the dermis and the adipose tissue.

### Microscopic characteristics of sections obtained after preliminary treatment with the body lotion

**Fig. 3:**
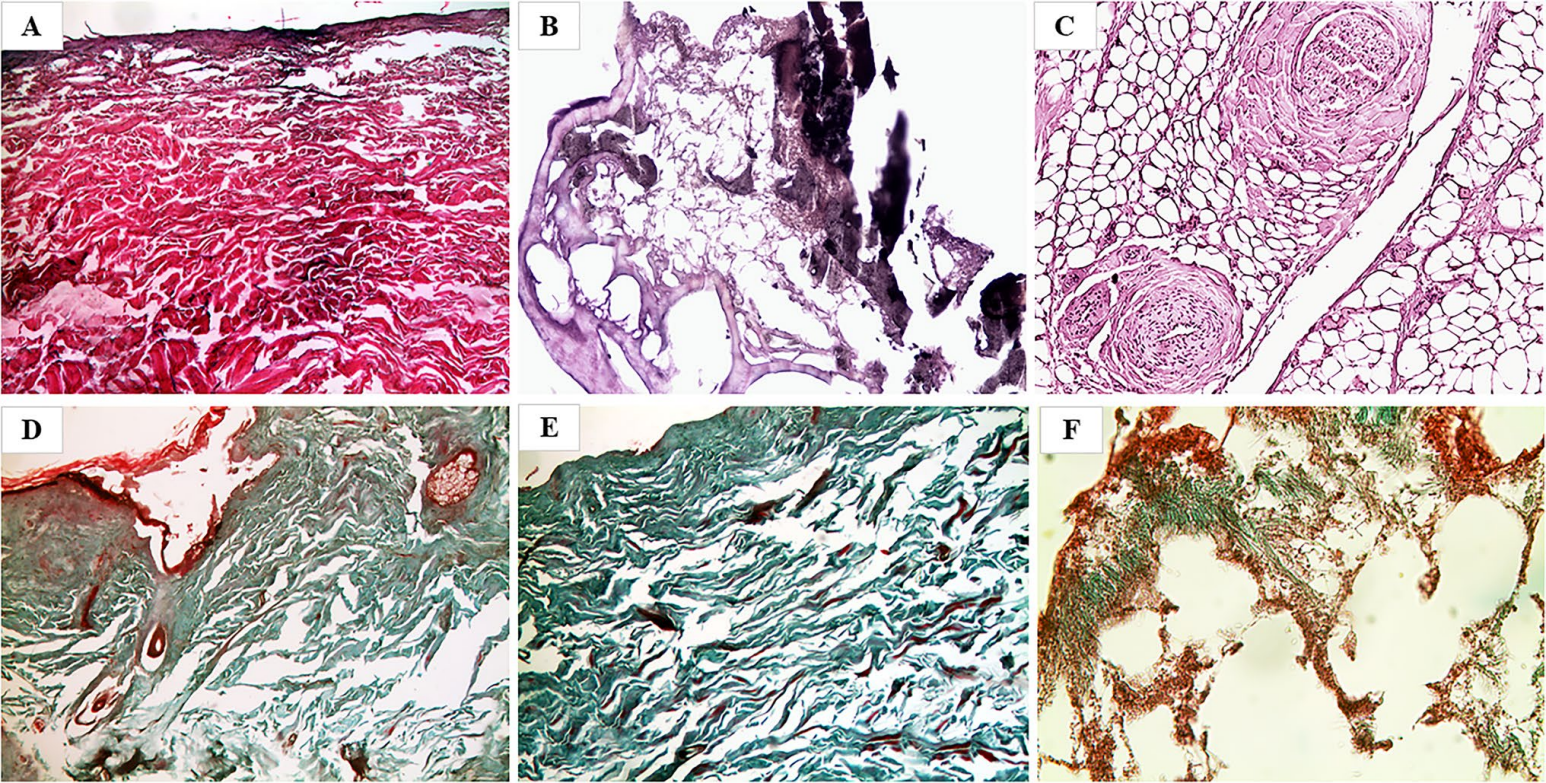
Microscopic view of preparations obtained after pretreatment with body lotion and then fixated in 4% formalin with the evidence of poor tissue restoration, permanence of some drying effects, and small connective areas still inverted and red stained. However, all histological structures are recognizable. **A**, **D** Case no. 17 (H&E, 50× and TM, 50× respectively). **E** Case no. 8 (TM, 50×). **B**, **C** Case no. 21 (H&E, 100× and 200×, respectively) with low restoration of the histomorphology, due to charring and corification. **F** Case no. 10 (TM, 400×), with suggestive areas of possible hemorrhagic micro-extravasation

All the samples showed a slight reduction in the destructive effects of the severe dehydration that were also observed in the samples directly fixated in formalin. In fact, most samples stained with H&E showed partial fragmentation (Fig. 3A), completely de-epithelialized portions, and rare areas of preservation of the epithelium layer. The keratin layer was frequently detached from the cellular epithelium layer, and numerous fungal and bacterial colonies were observed. Hair follicles with sebaceous glands were partially preserved both in their cellular and nuclear components. Some dermal vessels were dilated and filled with clotted blood; the skin elastic component appeared poorly preserved and, only in some cases, recognizable, thanks to the intense basophilic staining with H&E. In the specific case of mummified and partially charred skin (case no. 21), the staining with H&E showed the low efficacy of the body lotion in restoring the histological architecture (Fig. 3B, C). Sections were characterized by large areas of carbonization, protein coagulation, and marked loss of different skin portions; stainability was intensely basophilic, with a black-brownish coloration. Even the TM-stained twin sections showed a slight histological improvement (Fig. 3D) and a partial restoration of the stain affinity since only small areas of red (abnormal) rather than green (normal) coloration were still present (Fig. 3E). In case no. 10, the sample analyzed and pretreated with body lotion did not allow to evaluate evident hemorrhagic infiltrates, but only areas suggestive of possible hemorrhagic micro-extravasation localized in the subcutaneous adipose tissue (Fig. 3F).

### Microscopic characteristics of the slides obtained after preliminary treatment with fabric softener

**Fig. 4:**
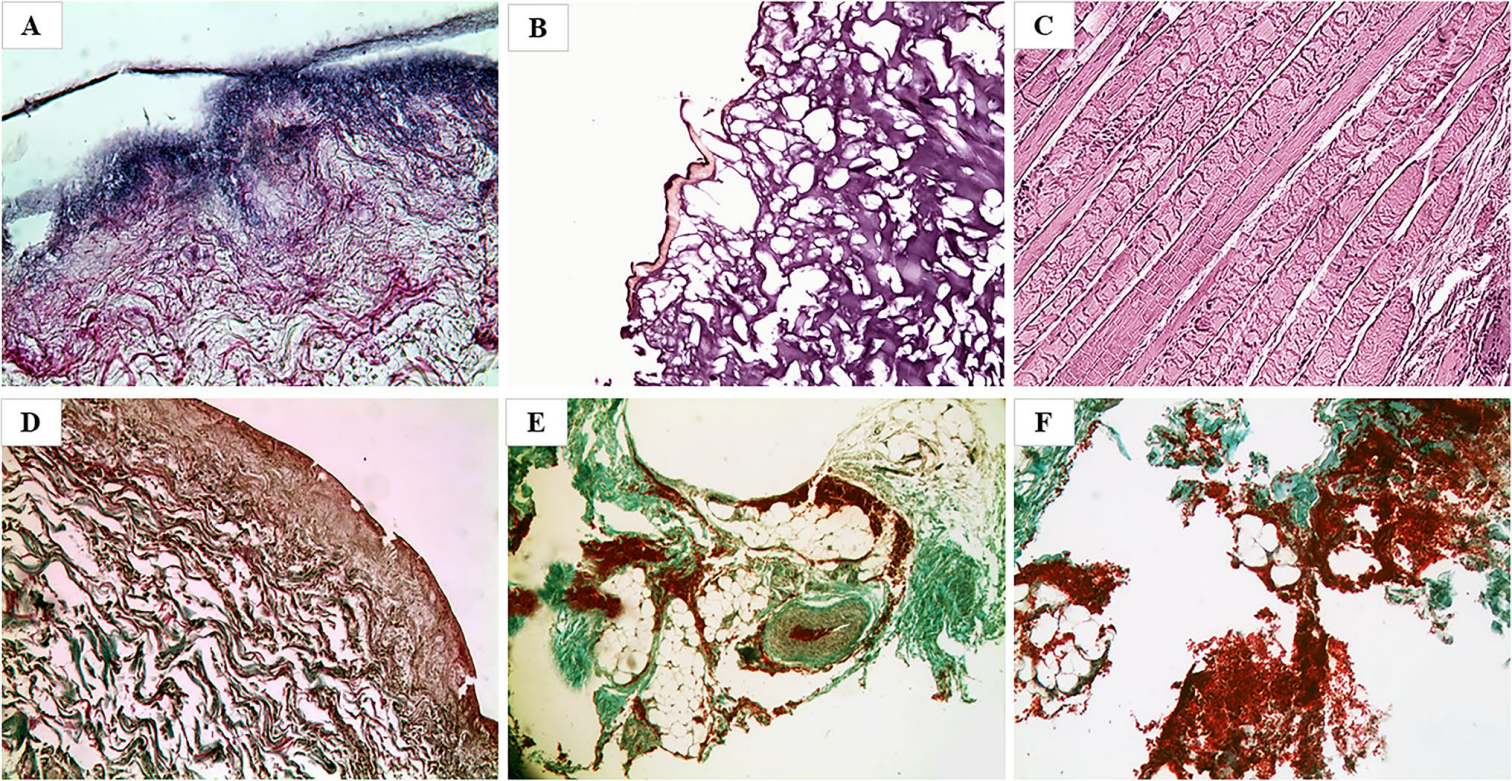
Microscopic view of slides obtained after pretreatment with the fabric softener and then fixated in 4% formalin with the evidence of a considerable reduction of destructive effects, better preservation of different tissue portions, and restoration of tissue stainability with the persistence of minimal inverted color areas. **A** Case no. 24 (H&E, 400×). **D** Case no.15 (TM, 100×). **B**, **C** Case no. 21 (H&E, 50× and 100×, respectively), in which an improvement of the histoarchitecture is observable with the evidence of heat injury signs both on skin and muscle. **E**, **F** Case no. 10 (TM, 50× and 100×, respectively) showing clear vital hemorrhagic infiltrates

All the samples showed better preservation of different skin portions and a significant reduction of the destructive features observed in the samples directly fixated in formalin. The skin fragment stained with H&E appeared only focally de-epithelialized, with scanty remnants of basal epithelial cells colonized by filiform fungal colonies and some spores (Fig. 4A). Occasional recognition of corneous material, hair follicles, and residues of small vascular and nervous branches in the subcutaneous adipose tissue was observed. In the specific case of mummified and partially charred skin (case no. 21), the staining with H&E demonstrated that the pretreatment with the fabric softener preserved large areas of charring of both epithelium and dermis, with an only partial detachment of dermal connective tissue and muscle fibers, protein coagulation, and altered black-brownish coloration (Fig. 4B); in the small and rare striated muscle flaps still persisting, multiple contraction bands and less impaired stainability were observed (Fig. 4C). Overall, therefore, pretreatment was also able to preserve the distinctive features of heat injuries. Twin sections stained with TM showed similar histological features and considerable restoration of the stain affinity since only minimal areas of red (abnormal) rather than green (normal) tissue coloration were still present (Fig. 4D). In case no.10, the sample analyzed and pretreated with fabric softener showed vital hemorrhagic foci, with clearly recognizable red blood cells in the subcutaneous adipose tissue, dissociating the structures in which they were extravasated (Fig. 4E, F).

### Microscopic characteristics of sections obtained after preliminary treatment with Sandison’s rehydrating solution

**Fig. 5:**
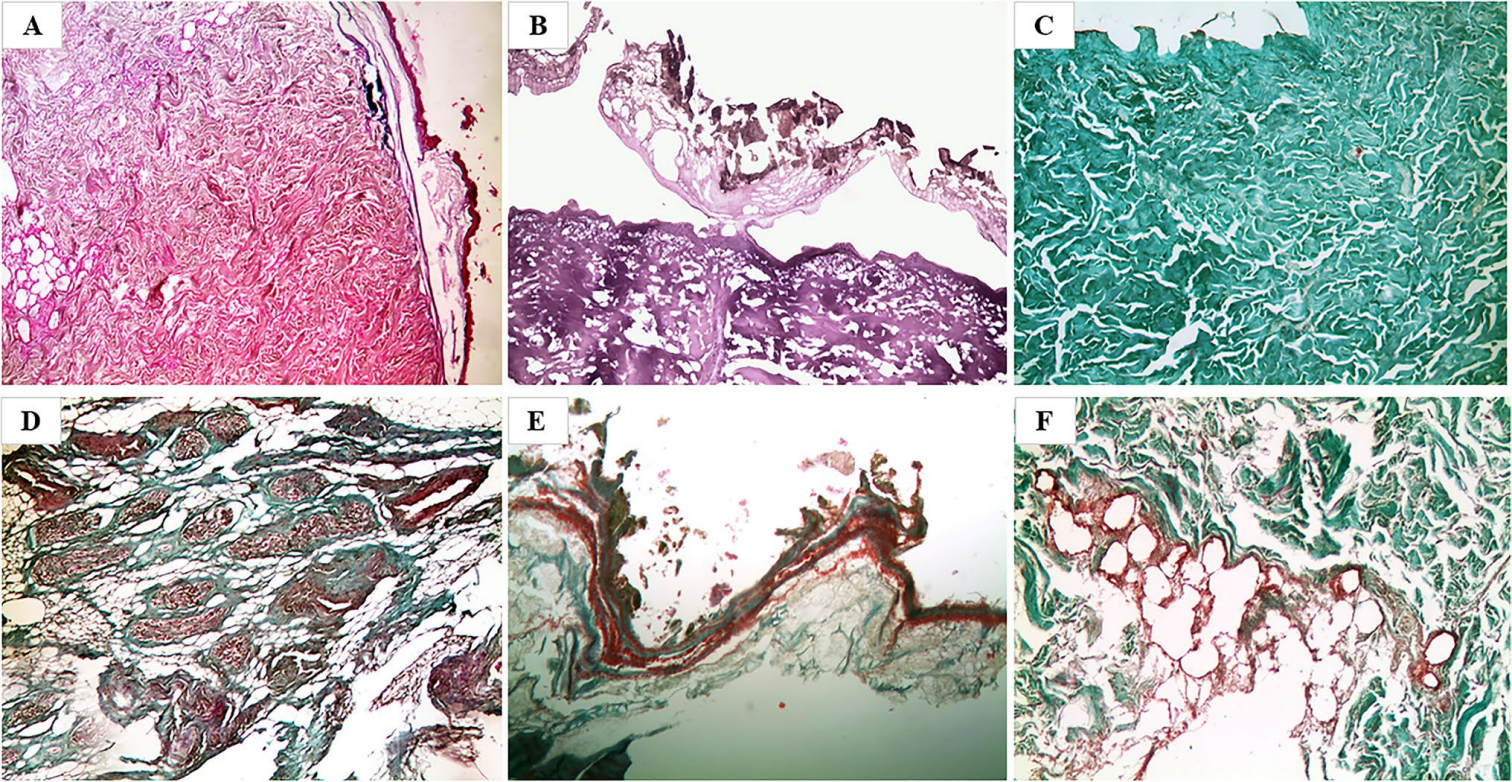
Microscopic view of preparations obtained after pretreatment with the Sandison and then fixated in formalin 4% with the evidence of a clear improvement and considerable reduction of structure alterations and artifacts with the complete restoration of the connective tissue stainability. **A** Case no. 3 (H&E, 50×). **C** Case no. 13 (TM, 50×). **D** Case no. 19 (TM, 50×). **B** Case no. 21 (H&E, 50×) with the evidence of ameliorative effect although with the evidence of heat injury signs. **E**, **F** case no. 10 (both TM, 50×) with the evidence of vital hemorrhagic infiltrates

All the samples showed a significant reduction of the stainability alterations compared to those observed both in the samples directly placed in formalin and placed in the other tested rehydrating substances. In fact, the skin fragments stained with H&E showed optimal histomorphological preservation, being documented by a better tissue homogeneity, compactness, and recognizability of histological structures. A better-preserved morphology of the whole tissue was generally observed, particularly regarding connective tissue and vessels; both elastic fibers and collagen were clearly detected (Fig. 5A). In the specific case of mummified and partially charred skin (case no. 21), H&E staining demonstrated that pretreatment with Sandison’s solution resulted in a marked general improvement in tissue morphology, with epithelium partly preserved and well recognizable (Fig. 5B). Overall, therefore, pretreatment preserved even the peculiar features of heat injuries. Twin sections stained with TM showed complete recognizability of the different skin layers and total restoration of the stainability of the connective tissue, which resulted in entirely green colored instead of red (Fig. 5C, D). In case no. 10, the sample analyzed and pretreated with Sandison’s solution presented an optimal histomorphology restoration (Fig. 5E), yet it was not possible to evaluate with certainty the presence of vital hemorrhagic infiltrates (Fig. 5F).

The main histological findings are reported in Table 2.

**Table 2:** Schematic representation of the main macroscopic and microscopic findings

Findings	Skin appearance		Direct fixation in formalin 4%	Preliminary treatment			Fixation in formalin 4% after the preliminary treatment
				Body lotion	Fabric softener	Sandison’s solution	
Macroscopic	Mummified	Hard consistency, parchment-like, and brownish-yellow colored	Rigid, retracted, with a very hard consistency, but also fragile and with an easy detachment of dry portions	Smooth, stretched, silky, and with a soft consistency (+)	Smooth, stretched, silky, and with a soft consistency (++)	Smooth, stretched, silky, and with a soft consistency (+++), and removal of the brownish pigmentations	Stiffening and hardening of skin pretreated with body lotion; no changes in skin with the other pretreatments
	Corified	Hard-elastic consistency and grayish colored					
Microscopic	Mummified		Marked deterioration effects and altered stain affinity due to the severe desiccation (+++)	Slight reduction of the destructive effects (++) and partial restoration of stain affinity	Important reduction of the destructive effects (+) and almost total restoration of stain affinity	Total reduction of the destructive effects and restoration of stain affinity	
	Corified						

**Fig. 6:**
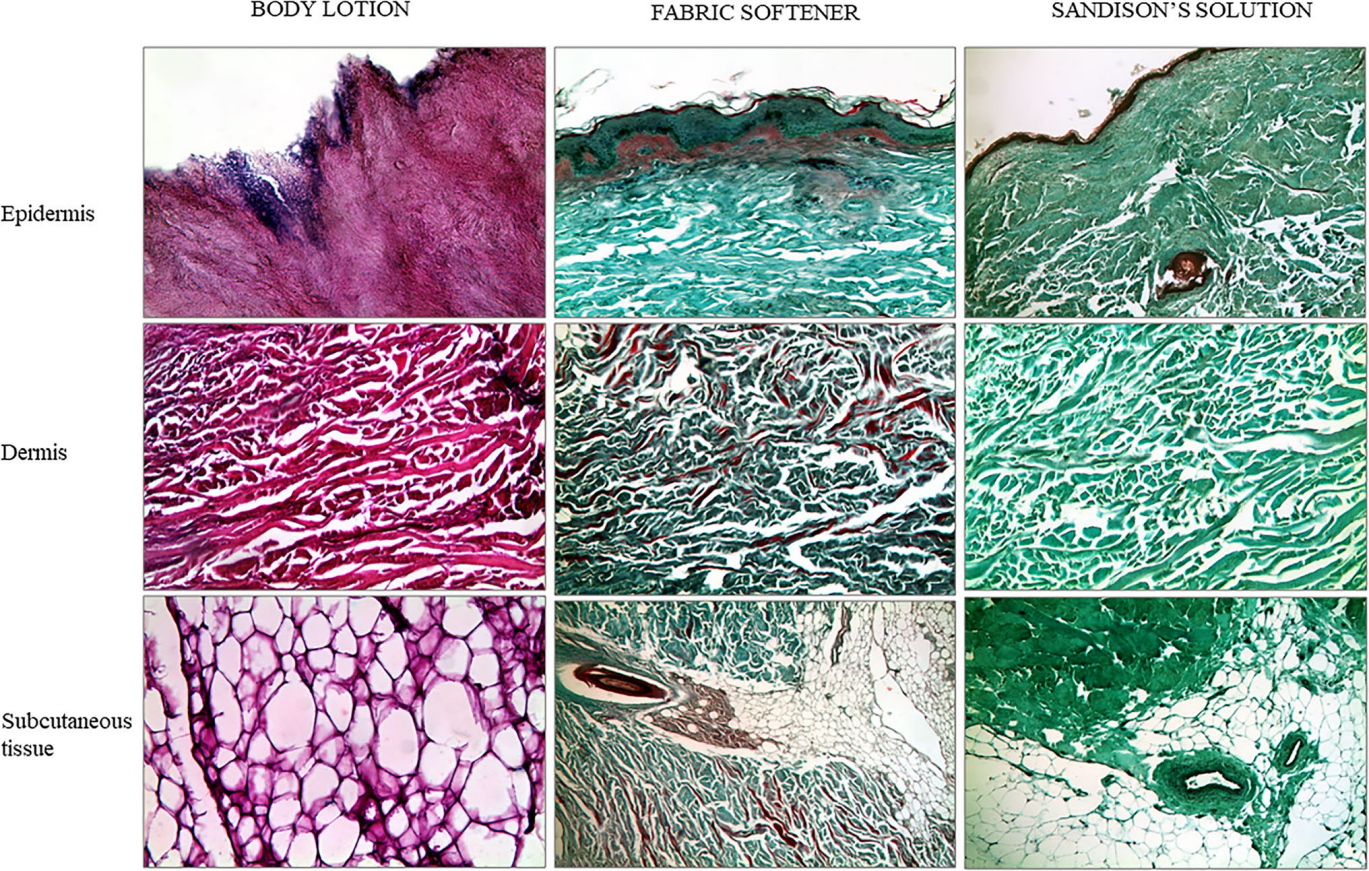
Comparative picture of the histological findings per specific skin layer that were documented after the three different pretreatments

To allow an easier comparison between the histological findings induced by the three different pretreatments, we have reported some significant images in a single table: every column represents a subgroup of pretreatment, every line a specific skin layer (Fig. 6).

## Discussion

Autopsy performed on mummified and corified corpses is characterized by considerable risk of misinterpretations [11], histological artifacts [12], and failure in determining both cause [13] and mode of death [8]. Indeed, since these tissues are both tough and fragile [14], as well as affected by severe structural alterations, many negative effects can be experienced in all the phases of the histological preparation [15]. First of all, the amount of biological matrices to be used in case of repetition of the examination due to sampling or diagnostic errors is very low [16]. There is also the real risk of the unfeasibility of identifying tissue structures and pathological conditions [8]. Despite these problems, literature [6] recognizes the great value and usefulness of the autopsy on mummified and corified bodies [7], as a gold standard to provide crucial information about injuries suffered by the victim or diseases that occurred intra vitam, even after many years [17–19].

Forensic histological studies focusing on human bodies in an advanced state of decomposition are few [4, 9, 20–24]. There are neither detailed protocols suitable for mummified or corified bodies nor consensus on the exact mechanisms involved in rehydration techniques [24]. These special post-mortem phenomena have been studied outside forensic practice only concerning a few aspects: rehydration with Sandison’s rehydrating solution to improve histoarchitecture [9], thermal stabilization to avoid its loss during preparation [25], prolongation of histological processing time for structural preservation [26], and successful detection of nuclear chromatin and DNA profiles in highly degraded cadavers without necessarily using bone tissue [27].

In mummified and corified cadavers, tissue-rehydrating techniques are essential to optimize the microscopic examination [28], allowing also to improve tissue staining affinity. In detail, they are able to remove the so-called paradox effect, i.e. the tendency of markedly dehydrated connective tissue to turn red (abnormal staining) instead of green [29, 30].

For these reasons, in the forensic field, there is a continuous quest for improving techniques to obtain high-quality microscopical preparations to facilitate and optimize diagnostics. Therefore, we tested the efficacy on mummified and corified human skin of two innovative commercial substances, i.e., a body lotion and a fabric softener. Their effectiveness was compared with direct fixation in formalin and with Sandison’s rehydration solution, which is currently considered in the literature as the best choice [9]. Our aim was to evaluate which rehydration solution performed best and whether there was a valid alternative to Sandison.

From a macroscopic point of view, we observed that after 3 days of pretreatment with body lotion, fabric softener, and Sandison’s rehydrating solution, the skin samples appeared to be softer than they were at the time of collection. Nevertheless, after standard fixation in 4% formalin, this characteristic was almost completely lost only in the case of pretreatment with the body lotion, resulting in not being very different from those directly fixed in formalin.

Microscopically, the samples preliminarily treated with the three different rehydrating solutions showed an improvement of the tissue structure and stainability compared to those directly fixated in formalin.

The body lotion, containing in it formulation of polyethylene glycol (PEG), acted as an emollient operating an initial softening and hydration of the skin tissue; moreover, thanks to the presence of silicone oils, it fluidized and made the skin smooth and silky, stretching it. However, these effects were unable to prevent the stiffening and coarctation produced by the subsequent fixation in 4% formalin. Therefore, histo-architectural restoration was only partial for both mummified (including partially charred) and corified skin.

The fabric softener, containing biodegradable cationic surfactants, was most likely able to penetrate deep into tissues, creating electrostatic charges able to distance the fibers from each other. This effect has prevented them from agglomerating, overlapping, and fragmenting, making the skin persistently softer and moisturized, and determining a wide restoration of normal staining. On the whole, there has been a high-grade restoration of the histological morphology for both mummified (including partially charred) and corified skin. Moreover, due to an antibacterial-antifungal agent among its constituents, this solution also prevented the microbial colonization of the skin.

Sandison’s rehydrating solution, containing an emulsifying agent (sodium carbonate, Na_2_CO_3_), was able to reduce the superficial tension of the water and to permeate homogeneously the cadaveric material. In addition, the solvent (96% ethanol) rehydrated and eliminated the effects of mummification/corification processes, giving stability to the tissues and preventing them from collapse. At last, the presence of the fixative formalin 1% had a bactericidal action that made the substrate not suitable for bacterial colonization and proliferation. Through this triple action, Sandison’s solution spread quickly and uniformly in the dry and friable skin, removing the dark-brownish pigmentation, giving the tissue persistent softness and transparency. Sandison’s rehydration solution was, therefore, the best solution compared to the other two tested substances.

Comparing the grade of morphological and staining restoration of the three tested substances, it emerges how the body lotion has operated a medium–low-grade restoration, and the fabric softener a high-grade restoration, while Sandison’s rehydration solution an optimal-grade restoration. We can clearly state that Sandison’s rehydration solution is the best rehydrating substance for mummified and corified skin, whereas the fabric softener could be considered a valid alternative.

In the particular case of no. 10, concerning a stabbing homicide with subsequent charring and mummification of the victim, in which the skin sampling was performed close to the edge of the wound, we observed a potentially significant fact that should not be underestimated. Indeed, surprisingly, the microscopic presence of vital hemorrhagic evidence was detectable only in the samples directly fixated in formalin and pretreated with the fabric softener. On the contrary, in the specimens pretreated with body lotion, there were areas suggestive of possible hemorrhagic micro-extravasation, while in the one pretreated with Sandison’s rehydrating solution, the presence of any element referable to blood extravasation could not be detected. It is therefore conceivable that different rehydrating substances may have a different impact on preserving the hemorrhagic infiltrate in the skin. In particular, as far as Sandison’s solution, the presence of Na_2_CO_3_ can cause the osmotic lysis of red blood cells [31, 32]; if confirmed, this could undoubtedly be a limitation for the application of Sandison’s solution, since it could deprive the forensic pathologist of crucial information, especially about the vitality of mummified or corified skin lesions or associated with underlying bone fractures. Therefore, further studies on the interaction between rehydrating substances and hemorrhagic infiltrate are necessary.

Meanwhile, this study has confirmed that Sandison’s rehydrating solution is the best performing rehydrating solution but fabric softener is unexpectedly able to provide a high-quality microscopic yield. This has been documented on both mummified (including partially charred) and corified tissues, proving to be extremely conservative also regarding hemorrhagic infiltrates. Therefore, in the absence of Sandison’s rehydrating solution, pretreatment with fabric softener could undoubtedly represent an excellent alternative.

## Data Availability

All the data have been reported in the manuscript.
